# *In situ* electrical and thermal monitoring of printed electronics by two-photon mapping

**DOI:** 10.1038/s41598-017-03891-7

**Published:** 2017-06-19

**Authors:** Francesco Pastorelli, Nicolò Accanto, Mikkel Jørgensen, Niek F. van Hulst, Frederik C. Krebs

**Affiliations:** 1 Department of Energy Conversion and Storage, Technical University of Denmark, Frederiksborgvej 399, DK-4000 Roskilde, Denmark; 2 ICFO - The Institute of Photonic Sciences, The Barcelona Institute of Science and Technology, 08860 Castelldefels (Barcelona), Spain; 3 ICREA - Institució Catalana de Recerca i Estudis Avançats, 08010 Barcelona, Spain

## Abstract

Printed electronics is emerging as a new, large scale and cost effective technology that will be disruptive in fields such as energy harvesting, consumer electronics and medical sensors. The performance of printed electronic devices relies principally on the carrier mobility and molecular packing of the polymer semiconductor material. Unfortunately, the analysis of such materials is generally performed with destructive techniques, which are hard to make compatible with *in situ* measurements, and pose a great obstacle for the mass production of printed electronics devices. A rapid, *in situ*, non-destructive and low-cost testing method is needed. In this study, we demonstrate that nonlinear optical microscopy is a promising technique to achieve this goal. Using ultrashort laser pulses we stimulate two-photon absorption in a roll coated polymer semiconductor and map the resulting two-photon induced photoluminescence and second harmonic response. We show that, in our experimental conditions, it is possible to relate the total amount of photoluminescence detected to important material properties such as the charge carrier density and the molecular packing of the printed polymer material, all with a spatial resolution of 400 nm. Importantly, this technique can be extended to the real time mapping of the polymer semiconductor film, even during the printing process, in which the high printing speed poses the need for equally high acquisition rates.

## Introduction

The performance of printed electronic devices relies principally on the characteristics of the polymer semiconductor material^1–3^. The analysis of such materials is generally performed with destructive techniques, which unfortunately are hard to make operational with *in situ* measurements^4–6^. A rapid, *in situ*, non-destructive and low-cost testing method is needed. In this study, we demonstrate that nonlinear optical microscopy is a promising technique to achieve this goal. Using ultrashort laser pulses we can map and monitor the charge carrier density and the molecular packing of the printed polymer material and we suggest that this technique can be extended to the real time mapping of the polymer semiconductor film while printed. We anticipate that this non-linear optical method will substantially contribute to the understanding of printed electronic devices and constitutes a promising novel tool, for non-destructive and facile testing of materials. This will help the production and development of high quality printed technologies, such as solar cells^7^, displays^8^ and sensors^9^.

The most important characteristics of a polymer semiconductor material are the charge carrier density, carrier mobility and molecular packing that are directly related to the morphology of the active material^10, 11^. However, a non-invasive, easy to implement and versatile characterization method capable of giving fast access to the spatially resolved material information is yet to be found. Atomic force microscopy is a well known tool to investigate the surface of a polymer, but it has the disadvantage of long scanning session and its invasive tip has to be replaced for each scanned sample^12, 13^. X-ray analysis are also well established tools for analysing the first few layers of material, but they also lack sampling speed and they work at best with highly ordered organic semiconductors. The sample is typically in vacuum, to avoid noise, or in helium environment, to avoid damaging^12, 14, 15^. More qualitative analysis can be made with thermography, optical imaging or luminescence that can give overall evaluation, on a more macroscopic scale, of the performances of the photoactive layer only^13, 16, 17^. In this work we show that confocal nonlinear optical microscopy, in which an ultrashort (20 fs) near infrared (central wavelength ~800 nm) laser pulse probes the sample, is a valid method to map the printed material with the minimal perturbation and the maximal spatial resolution (both in plane and in the z direction) obtainable with standard optical techniques. As the most common active materials for printed electronics only poorly absorb light at near infrared wavelengths, this choice greatly limits the sample photo-damage while increasing at the same time the penetration depth and the signal localization in the device compared with linear photoluminescence^18^. The increase in penetration depth could for instance allow researchers to probe multilayer configurations that are common in solar cell structures. The use of ultrashort laser pulses, on the other hand, concentrates all the available energy in very short light bursts that reduce to the minimum any laser-induced thermal effect and heating of the sample, while at the same time increasing the efficiency of nonlinear signals. As demonstrated in this work, our technique gives us access to different types of non-linear signals, which allows us to differentiate between the different materials of a printed device. This method is therefore very promising for non-invasive characterization of printed electronics devices and for many different materials.

**Figure 1:**
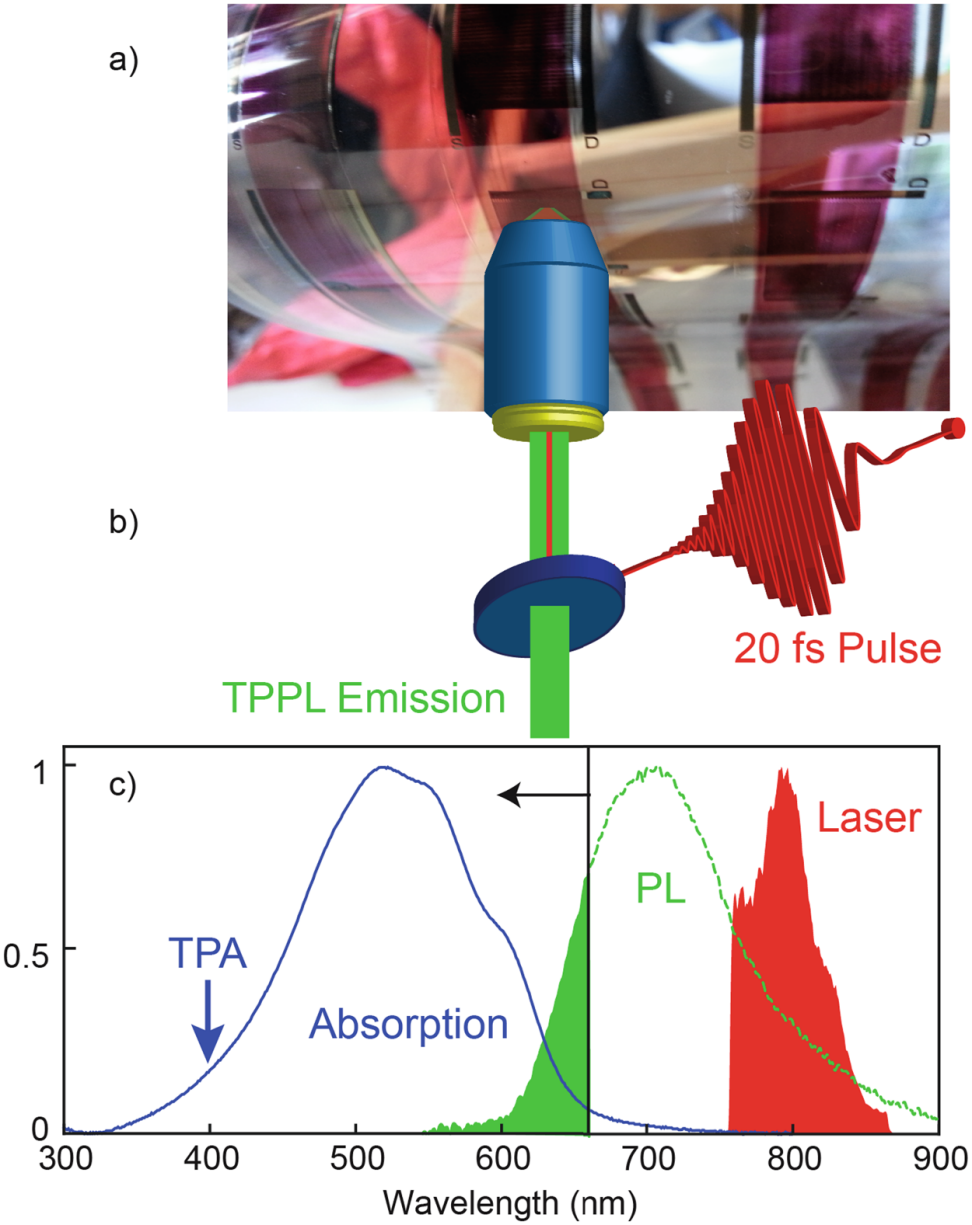
Non-linear optical characterization of a polymer semiconductor material roll coated on a flexible substrate. (**a**) Image of the flexible PET substrate with R2R printed source and drain silver electrodes covered by P3HT polymer semiconductor stripes. (**b**) Schematic of the non-linear optical microscope. (**c**) Absorption spectrum of P3HT (blue curve), emission spectrum of P3HT (green curve) and excitation laser spectrum (red coloured region). The green coloured region represents the amount of photoluminescence from P3HT detected in the experiment, considering that a 670 nm short pass filter is used. The blue arrow represents the effective wavelength at which the two-photon absorption occurs.

In our experiments, we stimulate two-photon absorption (TPA) in a roll coated polymer semiconductor (P3HT) and map the resulting two-photon induced photoluminescence (TPPL) and second harmonic (SH) response. First, we show that the different nonlinear optical signals can be used to discriminate between the polymer semiconductor material and embedded nanoparticles which constitute the electrode in a real device. Next we demonstrate that the TPPL quenches when applying a current between source and drain; this decrease can be used to determine the electrical characteristic of the device. Finally, we show that the total TPPL signal increases with higher temperature in the 20–120 °C range, closely following the supported current characteristics of the semiconductor. Because both the supported current and the optical properties depend on the charge carrier density and the molecular packing of the printed material^19^, we propose that the TPPL signal, at least in the experimental conditions used in this work, can be used to infer fundamental material properties in these devices. Importantly, simple calculations based on the signal levels, suggest that this technique can be extended to the real time mapping of the polymer semiconductor film, even during the printing process, in which the high printing speed poses the need for equally high acquisition rates. In Fig. 1, we show an image of the device investigated, namely a roll printed open transistor on a plastic flexible substrate (Fig. 1a), together with a schematic of the optical setup used for the characterization of the sample (Fig. 1b) and a graph showing the optical spectra of relevance for the experiment, i.e. the laser spectrum, the absorption and emission spectra of P3HT (Fig. 1c).

Source and drain electrodes of the open transistor were roll-to-roll (R2R) flexo printed on PET foil at a web speed of 20 m min^−1^ using an anilox volume of 1.5 mL m^2^. The water-based silver (Ag) nanoparticle ink (PChem PFI-722) was dried and sintered using hot-air (140 °C) and infrared lamps during printing. Further surface treatment, without major effect on the conductivity, was carried out using photonic flashlight sintering (Xenon Sinteron 2000). P3HT was annealed at 60 °C for 10 min and a dielectric capping layer was annealed at 120 °C for 30 min. Both layers were slot-die coated on a mini roll coater to simulate R2R environment. All processes were carried out at ambient conditions.

The laser source in the experiment is a ~20 fs titanium sapphire laser (Octavius 85 M, Menlo Systems) tuned to a central wavelength of ∼800 nm with a bandwidth of ∼70 nm (see spectrum in Fig. 1). A pulse shaper based on a liquid crystal spatial light modulator is used to compress the laser pulse in time at the sample position^20^. The laser pulse is then sent to a two-photon confocal microscope equipped with a high numerical aperture (0.95) air objective that ensures high spatial resolution. The objective focuses the laser beam on the P3HT layer through the PET layer. The sample can be raster scanned with respect to the objective within an area of 100 µm × 100 µm using a nanometre precision piezoelectric scanner. Nonlinear optical signals such as TPPL and SH emitted by the sample, are then collected by the same objective in reflection geometry, spectrally filtered to reject the reflected laser and sent to the photodetectors. Two-dimensional images of the sample are acquired using an avalanche photodiode (APD) as photodetector while raster scanning the sample, whereas a spectrometer equipped with an electron-multiplying charge-coupled device camera is used for spectral measurements. As the spectra of Fig. 1 show, in the experiment we use a 670 nm short pulse optical filter to reject the laser light and collect nonlinear signals emitted at shorter wavelengths. The P3HT is excited through a TPA process (therefore at the effective wavelength of 400 nm) and the blue tail of the emitted TPPL is detected.

**Figure 2:**
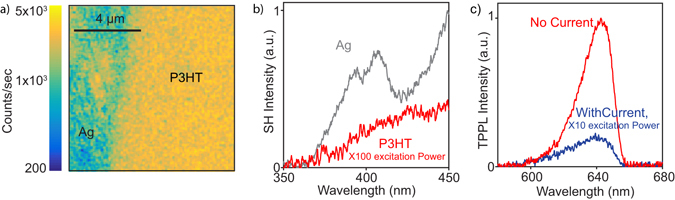
Non-linear photoluminescence. (**a**) TPPL spatial map at the interface between Ag and P3HT. (**b**) SH spectrum from the Ag electrode. The double peak in the spectrum is likely due to a non perfectly compressed pulse, as explained in ref. 20. In the same wavelength range optical signal from P3HT can only be detected upon two orders of magnitude increase in the excitation power. (**c**) Two photon photoluminescence spectrum from P3HT polymer with no current (red curve) and with current (blue curve) applied. The spectra are cut at 670 nm by the short pass filter used in the measurement. The application of a current strongly quenches the TPPL signal.

In Fig. 2a we show a two dimensional optical image of the device acquired by scanning the sample on a 10 µm by 10 µm area with a precision of 50 nm while continuously exciting it with the ultrashort laser pulse and detecting the nonlinear emission (at wavelengths below 670 nm) with the APD. The spatial resolution of the image (limited by diffraction) is about 400 nm. Two different domains can be distinguished based on the detected signal: a low intensity one on the left side, attributed the Ag electrode, and a high intensity one on the right side, attributed to the P3HT. When spectrally resolving the detected light, the two domains are found to have different spectral signatures. The Ag electrode produces both a very broad TPPL spectrum, extending from 450 nm to more than 670 nm and a much narrower SH spectrum around 400 nm, in agreement with previous findings on Ag nanoparticles^21, 22^. In contrast, P3HT mainly emits TPPL, which, when using a 670 short pass optical filter, results in a relatively narrow emission around 640 nm. The SH from Ag and the TPPL from P3HT can thus be used to discriminate between the two materials, and more generally between the P3HT and any material with a defined SH signature, which is an advantage of the nonlinear optical technique used not achievable with single photon photo-luminescence or other qualitative measurements^17^. In the graph of Fig. 2b, we show the SH spectrum from the Ag electrode, which indeed forms a well-defined peak centred at 400 nm. Instead, the very low signal at these wavelengths emitted by P3HT is only observable when increasing the excitation power by two orders of magnitude (from 100 µW to 10 mW, that correspond to a power density of 0.1 to 10 MW/cm^2^). The graph of Fig. 2c shows TPPL spectra from P3HT, which are cut around 670 nm, due to the presence of the short pass filter.

The two different curves in the graph of Fig. 2c correspond to the TPPL emission from P3HT acquired with and without the application of a current between source and drain. The transistor supported current is applied by connecting a power supply to the electrodes with a fixed voltage of 1 V. In a semiconductor, this has the effect of rapidly separating the photo-created charges (electron and holes), which no longer can recombine to produce photoluminescence. In the experiment indeed, upon application of a current the TPPL is highly quenched, as shown in the graph Fig. 2c. The integrated TPPL signal, in the case of an applied current, is ~4 times lower than the one measured for zero current and is obtained for an incident power 10 times higher. Considering that the TPPL scales as the square of the excitation power, we can estimate a decrease in the TPPL intensity of a factor of ~400 upon application of a current. The current ON/OFF experiment demonstrates that carrier density and TPPL efficiently can be related in an *in situ* measurement. This confirms that the device works electrically and that our nonlinear optical characterization is well suited to test the electrical properties of printed electronics devices.

The electron/hole mobility in a solid-state semiconductor, is only marginally affected by temperature. Organic semiconductors in contrast, can present a stronger relation between mobility and temperature^19, 23, 24^. Devices show different characteristics in relation to the working environment temperature. High molecular weight P3HT transistors have shown the best performances in terms of mobility at a temperature of 90 °C^24^, an effect mostly due to the interchain overlap change of the P3HT. At the same time, the optical properties of these materials also depend on the molecular packing and hence on the working temperature^19, 25^.

**Figure 3:**
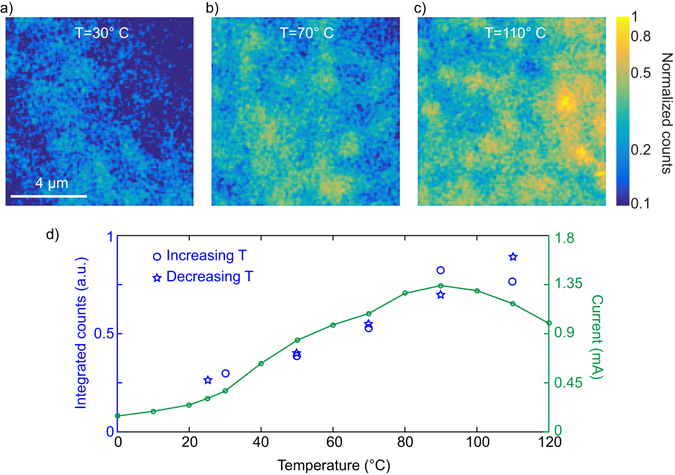
Temperature evolution of the two photon photoluminescence. (**a**–**c**) TPPL spatial map of P3HT at different temperatures, with a diffraction limited resolution (~400 nm). (**d**) Temperature characteristic of the two-photon photoluminescence for increasing temperature (blue circles) and decreasing temperature (blue stars) and of the supported current (green curve) in the polymer semiconductor transistor. The error bar in the measurement of the TPPL, obtained as the sum in quadrature of the error of each pixel in the spatial images (calculated as √N, where N is the TPPL signal at each pixel), is always smaller than the size of the symbol used.

We show here that, by introducing an experimental way to *in situ* vary and measure the temperature of the semiconductor, we can deterministically change its morphology in terms of molecular packing and hence its charge carrier density. This in turn strongly influences both the supported current and the TPPL signal. The temperature of the open transistor is varied in the range 20–120 °C with a Peltier cell controlled in current and tuned by a Fluke thermal imager Ti 125 with an accuracy of ±2 °C +2% of the temperature in °C. For every applied temperature, we acquire two-dimensional TPPL images of the sample. Examples of images, corresponding to temperatures of 30, 70 and 110 °C, are shown in Fig. 3a–c. The data for the three images is normalized to the maximum of the 110 °C image. It is apparent that the TPPL signal increases for higher temperatures, which, for the colour scale used in Fig. 3a–c, results in brighter colours in the optical images. We note here that, when changing the temperature of the sample, as a consequence of thermal dilatation, the focus of the microscope objective has to be slightly adjusted to maximize the collected optical signal. In the graph of Fig. 3d we report the spatially integrated intensity from the two-dimensional optical images for every temperature and compare it to the measured supported current characteristic. The behaviour of the two quantities is very similar, which strongly suggests that the TPPL intensity, for this material and in the current experimental conditions, can be used as an indicator of the charge carrier density and molecular packing of the printed material. Moreover, the behaviour of the TPPL as a function of temperature is reversible, as for the supported current: when decreasing the temperature form 110 °C to room temperature, the TPPL decreases to its minimum value, as demonstrated in Fig. 3d.

The temperature dependence of the supported current can be understood as follows: at temperature between 0 °C and 20 °C the activation energy needed to promote the carrier in a conduction state is the highest, owing to lower charge mobility, shorter P3HT chain length and other effects^26–31^. Here the supported current and TPPL are at their lowest. For temperature between 20 °C and 90 °C the supported current and the TPPL increase linearly with the temperature, as a consequence of an interchain overlap change^19^ and a reduced activation energy that improves the overall mobility. The material is efficiently packed and has a better intermolecular overlap^25^. This allows a better hopping of charges between molecules and thus an improvement in the mobility.

The fact that the TPPL intensity follows the same behaviour as the supported current clearly points towards a common origin for the temperature dependence of both processes, and justifies, at least in this case, the use of the TPPL as an indicator of the molecular packing and mobility. This phenomenon can be qualitatively understood by considering that, as shown in ref. 19, for high molecular weight P3HT, the molecular packing influences the absorption cross section of the material. Our experiment is in turn very sensitive to changes in the absorption cross section, as they directly impact the total TPPL signal. From the variation of the TPPL signal in a given area of the sample, we can therefore deduce the molecular packing state of the material by using the graph in Fig. 3d.

Above 90 °C the disorder contribution^25^, the increase of various resistance effects^32^ and the decrease of interchain transport^33^, due to the temperature, start to dominate resulting in a reduction of the supported current. In this range, the behaviour of the TPPL from the graph in Fig. 3c cannot be precisely inferred from the experimental points available. We find that the TPPL do not drop as the supported current do. With that we can hypothesized that the carriers, visualized by the TPPL increase and that the drop in overall current is due to the above mentioned decrease in interchain transport effects. Finally, we wish to highlight that our two-photon characterization is, at least for what it concerns the count level, applicable to the mapping of printed devices even during the high speed R2R device fabrication. Considering that typical fabrication speeds reach 20 m min^−1^, and that the TPPL counts we detected (see for example Fig. 2) is at the few K count s^−1^ level, an increase in the detected signal of about two orders of magnitude would result in about 10^6^ counts s^−1^, i.e. more than a million counts while a meter of material passes. A two order of magnitude increase in TPPL could easily be obtained by increasing the excitation power of a factor of 10, and/or by more effectively detecting the TPPL spectrum (e.g. by using a different short pass filter), or with a laser source producing short pulses at a wavelength around 1000 nm. This means that, even during high speed fabrication, our method already has the required sensitivity to resolve changes in the working temperature or in the molecular packing of the printed material averaged over 10 µm where other methods can only average over centimeters^4, 34^.

In conclusion, we have demonstrated that nonlinear optical microscopy is a powerful, versatile and non-destructive method to easily characterize printed electronics devices, with a spatial resolution down to 400 nm. In our case, the level of TPPL can be used as an indicator of molecular packing. This might be extended to other materials whose optical properties depend on the molecular packing, even if new experiments adjusted for each case will be necessary. We have shown that the different nonlinear signals generated by different components in the device can be used to discriminate between semiconductor and NPs. Subsequently, we have demonstrated that the TPPL emitted by the active material can be quenched by the presence of an applied current between source and drain. This is fundamental for *in situ* measurements where real devices can be electro-optically characterized with high spatial resolution. Most importantly, we have provided evidence for a relation between the molecular packing of the active material, carrier density and the TPPL intensity. By studying the temperature dependence of the TPPL and comparing it with the supported current in the device, we have shown that at 90 °C the semiconductor is characterized by a better interchain overlap and therefore higher mobility. This means that nonlinear optical microscopy is able to extract fundamental information about printed electronics devices, such as free charges and NPs contents and, when combined with electrical measurements, mobility and molecular interchain overlap *in situ* and in a completely non-destructive way. These information will allow printed electronics to emerge as a new, large scale and cost effective technology that will be disruptive in fields such as energy harvesting, consumer electronics and medical sensors.
